# Identifying and understanding the health and social care needs of Indigenous older adults with multiple chronic conditions and their caregivers: a scoping review

**DOI:** 10.1186/s12877-020-01552-5

**Published:** 2020-04-19

**Authors:** Sharlene Webkamigad, Robyn Rowe, Shanna Peltier, Amanda Froehlich Chow, Katherine S. McGilton, Jennifer D. Walker

**Affiliations:** 1 School of Rural and Northern Health, Laurentian University, Sudbury, Ontario P3E 2C6 Canada; 2 Ontario Institute for Studies in Education, University of Toronto, Toronto, ON Canada; 3 Canadian Centre for Health and Safety in Agriculture, University of Saskatchewan, Saskatoon, SK Canada; 4 Toronto Rehabilitation Institute - University Health Network, Toronto, ON Canada; 5 Lawrence S. Bloomberg Faculty of Nursing, University of Toronto, Toronto, ON Canada

**Keywords:** Scoping review, Multimorbidity, Older adults, Caregivers, Health care providers, Needs, Indigenous, Determinants of health, Well-being

## Abstract

**Background:**

Indigenous people continue to experience high rates of multiple chronic conditions (MCC) at younger ages than other populations, resulting in an increase in health and social care needs. Those who provide services designed to address MCC for Indigenous communities require synthesized information to develop interventions that meet the needs of their older adult population. This review seeks to answer the research question: What are the health and social care needs, priorities and preferences of Indigenous older adults (living outside of long-term care settings) with MCC and their caregivers?

**Methods:**

A scoping review, guided by a refinement of the Arksey & O’Malley framework, was conducted. Articles were included if the authors reported on health and social care needs and priorities of older Indigenous adults. We also included articles that focused on Indigenous conceptions of wellness, resilience, well-being, and/or balance within the context of aging, and articles where authors drew from Indigenous specific worldviews, ways of knowing, cultural safety, cultural competence, cultural appropriateness, cultural relevance and community needs.

**Results:**

This scoping review included 9 articles that were examined using an Indigenous determinants of health (IDH) theoretical framework to analyze the needs of older adults and CGs. Five areas of needs were identified: accessible health services; building community capacity; improved social support networks; preservation of cultural values in health care; and wellness-based approaches.

**Conclusion:**

The review highlights key determinants of health that influenced older adults’ needs: education and literacy, ethnicity, and social support/network (proximal); health promotion and health care (intermediate); and a combination of historical and contemporary structures (distal). The findings highlight the importance of local Indigenous knowledge and perspectives to improve accessibility of culturally relevant health and social services.

## Background

The aging of Indigenous populations is pressuring health and social care systems to recognize and account for the needs and cultural values within diverse Indigenous communities [1, 2]. Despite the continuing support and research into Indigenous experiences and efforts to age well [1], Indigenous people in countries such as Canada, the United States, Australia, and Aotearoa/New Zealand continue to experience high rates of multiple chronic conditions (MCC) at younger ages than other populations [1, 3, 4]; resulting in an increase in health and social care needs. Internationally, aging populations are increasing economic demands on services such as housing, public pensions, and health and long-term care supports that assist in aging well [1, 5]. This adds additional challenges for Indigenous people, families, and communities who must work to develop community-level supports in collaboration with settler-government partners [6].

Indigenous leaders and those who provide services designed to address MCC for Indigenous communities require synthesized information on emerging social and health care needs. Despite the need for both Indigenous specific and local information to guide effective planning, to our knowledge, there is no review of the health and social care needs of Indigenous older adults with MCC and their caregivers. As a starting point, we aimed to conduct a comprehensive scoping review asking the following research question: What are the health and social care needs, priorities, and preferences of Indigenous older adults (living outside of long-term care settings) with MCC and their caregivers?

As Indigenous peoples experience population aging, Indigenous leadership, researchers, and policy-influencers are making efforts to support communities by embracing that Indigenous determinants of health are distally rooted in historical and ongoing acts of colonialism [7]. These efforts are aimed at recovering and preserving the well-being, independence, and autonomy of Indigenous peoples. They also aim to understand the health and social care needs for older Indigenous people [1, 3, 8, 9]. This understanding led to the use of an Indigenous Determinants of Health (IDH) theoretical framework that is not purely ‘social’ in nature and does not overlook the ongoing impact of colonialism [7, 10, 11]. Further, this paper incorporates the International Classification of Functioning, Disability and Health (ICF) which was supported by the World Health Organization in 2001 and implemented here as a tool to offer a comprehensive perspective of the health and functioning experiences of Indigenous populations in an effort to interpret the results of the review [12].

The Indigenous Determinants of Health theoretical framework is best described by Charlotte Reading using the metaphor of a tree [7]. The IDH theoretical framework recognizes that the historical legacies within settler dominated societies have distal, deeply rooted, influences that have led to complex interconnected impacts to intermediate (trunk) and proximal (crown and leaves) determinants of Indigenous peoples’ health [11]. Like the tree, the interconnections between these three determinants rely on one another. Briefly, elements that begin at the distal impact the intermediate, resulting in advantages or disadvantages at the proximal level which ultimately impact the health and wellbeing of the individual. The distal determinants are reflective of things that impact health located externally to the individual [11, 13]. The roots of trees are far reaching and often unseen; through this representation, they capture the historical, political, ideological, economical, and social foundations that create the infrastructure for the intermediate and proximal determinants [11]. The deeply embedded roots of Indigenous worldviews, spirituality and self-determination are reflected through the historical and contemporary structures of the distal determinants [11]. An example of how distal determinants affect Indigenous peoples health is through colonialism, which has associated in social, material, and health inequities [11]. Intermediate determinants include things that impact the environment of the individual such as health care, education, social supports, and government resources [10]. Proximal determinants have direct impacts on physical, emotional, spiritual and mental health [10] and include early child development, income and social status, education and literacy, social support networks, employment, working conditions and occupational health, the physical environment, culture, and gender [13]. Disadvantages within these areas (through poverty, discrimination, lack of education, etc.) have shown to result in poorer health status across physical, emotional, mental spiritual, and social realms that effect individual wellness [11]. Nourished roots (distal) and a strong trunk (intermediate) can impact the health of the crown, or the individual. Employing an Indigenous framework aids in recognizing the importance of relationships, kinship, language, and cultural traditions as having a direct impact on the proximal determinants of the individual [11].

In addition to the IDH theoretical framework, the ICF framework offered a comprehensive, multidimensional, and holistic context at both the individual and population level. This framework implies that the interaction between an individual (with a health condition) and contextual factors (environmental and personal factors) lead to (a) impairments in body functions and structures, (b) limitations in activity, and (c) restrictions in participation [12, 14]. Combining the two frameworks assisted in understanding health and social care needs and preferences as related to wellbeing and quality of life on overall functioning [12].

## Methods

This scoping review was an Indigenous-specific sub-project of a broader review whose review protocol and results have been published [15, 16]. The scoping review protocol focused on understanding the health and social care needs of all community-dwelling older adults with MCC [15]. During the review process, important information was gained about the unique needs of Indigenous older adults with MCC. Thus, after consultation with Indigenous partners and researchers, it became evident that a separate Indigenous-focused review was necessary. This review acknowledges the significance of Indigenous community research ethics and perspectives on Indigenous determinants of health, therefore a sub-team of researchers (including the Principal Investigator (PI) of the overall project (KM) and Indigenous researchers) was created to conduct a review of the Indigenous-specific papers. We used the scoping review methods framework outlined by Arksey and O’Malley [17] and refined by Levac et al. [18], Colquhoun et al. [19], and Daudt et al. [20]. In addition, we followed the Preferred Reporting Items for Systematic Reviews and Meta-Analyses Protocol (PRISMA-P) [21] throughout the development and preparation of study protocols. As part of the sub-project, this scoping review included six steps: (1) Identifying the research questions (above); (2) identifying the relevant literature; (3) study selection; (4) charting the data; (5) collating, summarizing, and reporting the results; and (6) consulting with key stakeholders and translating knowledge.

Following the development of the research questions, two academic health sciences librarians prepared the search strategy in consultation with the original research team, which included one Indigenous researcher (JW). This comprehensive review involved multiple databases including OVID Medline (1946 to 2017, including Epub Ahead of Print, and In Process & Other Non-Indexed Citations), OVID Embase (1947 to 2017), OVID PsycINFO (1806 to 2017), OVID Social Work Abstracts (1968–2017), EBSCO CINAHL Plus with Full Text (1981 to 2017), EBSCO AgeLine (1966–2017), and Cochrane Central. The search strategies were translated using each database platform’s command language, controlled vocabulary, and appropriate search fields. The search concepts of health and social care needs and priorities, Indigenous populations, and multimorbidity employed the use of MeSH terms, EMTREE terms, APA thesauri terms, CINAHL headings, and text words. Specific search terms for Indigenous populations included those listed in Table S1: Medline Search.

We applied a modified adult age filter to the Medline strategy [22]. This filter was translated and applied to the Embase, PsycINFO, CINAHL, and Cochrane Central search strategies. The filters were not validated. Language limits were applied to capture articles in English, French, Dutch, and German, in all databases where applicable [15]; however only English papers were included in the Indigenous sub-analysis. Unfortunately, the original review methods were not designed to include Spanish or Portuguese articles, which may limit the applicability to Indigenous populations in Central and South America. In May 2017, the research team concluded the final searches. The full Medline strategy is included in Additional file 1: Table S1. Additionally, we searched the reference lists of included studies. Covidence systematic review software was used to facilitate the review [23].

Study selection followed a two-step process [15]: 1) an initial title and abstract review (KM, JW, AFC, RR, SW, SP), and 2) an independent review and assessment by Indigenous reviewers of potentially relevant full text articles for inclusion (SW, RR, SP, AFC). In case of disagreement between reviewers, the lead Indigenous researcher (JW) solved the conflict. Through a multi-step process, both published and unpublished literature was reviewed. The inclusion criteria included:

- Studies with any type of design, including quantitative, qualitative, mixed or multi-methods research, arts-based, and comparative methods designs were included for review.
- Reports on the health and/or social care needs, preferences, and/or priorities of Indigenous peoples, and/or caregivers of older Indigenous adults. This included both informal (family/friend) and formal (health care professional) caregivers who provided care for an Indigenous community member.
- Literature with a focus on Indigenous populations and persons with a mean age of 55 years and older or including a sub-group analysis for Indigenous persons living with MCC, their caregivers, or health care providers.
- Focus on wellness, resilience, well-being, and/or balance within the context of aging.
- Indigenous specific worldviews, ways of knowing, cultural safety, cultural competence, cultural appropriateness, cultural relevance and community needs.

The exclusion criteria included:

- Expert opinions, editorials and materials that did not include original data.
- Publications in languages other than English.

Data abstraction was completed using an Excel Spreadsheet. The data for each study was abstracted by two independent reviewers. Codes representing the World Health Organization’s (WHO) definition of structural and social determinants of health [24] combined with Loppie-Reading’s definition of the IDH [11] were included to map the studies that considered these as important considerations in identifying needs of older adults with MCC. Data extraction included: details on the study (aim of the study, study design, study location, etc.), study characteristics (setting, sample type, sampling method, source of data, etc.), patient and/or caregiver characteristics (age, gender, ethnicity, location, marital status, location [urban/rural/other], relationship to patient, chronic condition/other health information, etc.), socio-economic status of patient (employment, income, education, food security, etc.), health professional characteristics (speciality, experience, etc.), health and social care needs (how were they measured, actual needs identified, etc.), social support networks for patients and/or caregiver (family, neighbours, etc.), and early childhood development. In general, we used the word “Indigenous” as an inclusive term that refers to people who are Indigenous to their respective homelands. However, we retained the terminology that each paper used to refer to the Indigenous Nation involved in each of the included studies (i.e. First Nation, Aboriginal and Torres Strait Islander, Indigenous Australian, Native Hawai’ian, etc.)

The quality of the research studies was assessed using the Mixed Methods Appraisal Tool (MMAT) [25]. The MMAT allows inclusion of qualitative, quantitative, and mixed methods studies with quality criteria relevant to each study design. No studies were excluded based on the quality assessments. In addition, the following quality criteria were applied to assess alignment with established standards in Indigenous health research methods: 1) Indigenous community engagement, and 2) integration of Indigenous perspectives. The Indigenous community engagement criterion was met if the author list included co-authors from Indigenous communities or organizations or if there was explicit description of community engagement approaches. The Indigenous perspectives criterion was met if the research teams included (or consulted with) Indigenous Elders, Knowledge Keepers or both, established an Indigenous advisory structure, used an Indigenous theoretical framework, or employed Indigenous research methods.

To map the data and conduct a thematic content analysis, we used the two frameworks described above. Where appropriate, we used deductive thematic analysis on relevant extracted data from the published studies to map the studies. We aimed to incorporate a rigorous methodological approach suggested within the literature [18]. Consultation has been highlighted as an optional step within the chosen framework [17], therefore we drew on our extensive relationships within the Indigenous community to gather additional perspectives on the themes that were generated in the analysis. On October 10, 2018, we hosted a small, focused workshop that included First Nations community members, including those living with chronic conditions, health care providers, policy and research analysts working in First Nations political organizations, and academic researchers. We described the scoping review and discussed the themes that emerged from the analysis. Their feedback was included and was used to inform the results.

## Results

We present the results in two ways: 1) a numerical overview of the amount, type, and distribution of the included literature; and 2) a narrative synthesis and mapping of the results.

### Characteristics of the included studies

**Fig. 1:**
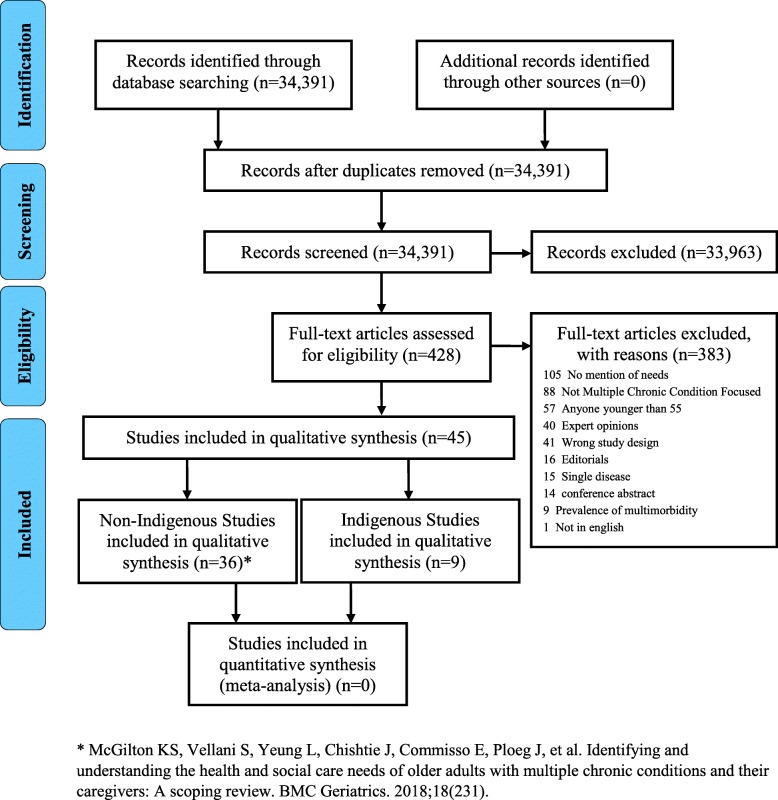
PRISMA Flow Diagram

Following the previously published protocol [14], 34,391 titles and abstracts were reviewed by two independent researchers [see Fig. 1. PRISMA Flow Diagram]. After removing the duplicates and adjusting for the inclusion decisions following the first phase of review, 428 manuscripts were selected for the full text review. The full text review removed 383 manuscripts, and the remaining 45 articles moved forward. Of these, nine were included in the Indigenous review [Table 1: Overview and description of included studies]. All the included manuscripts were written in English. Eight studies used qualitative methods [1–3, 8, 9, 26–28], and one used a cross-sectional design [6]. Five were conducted in Australia [1, 2, 8, 26, 28], while four were based in North America; including Hawai’i [3, 27], Northwestern Ontario [9], and the United States [6]. Studies examined the complexities, experiences and health care needs of aging Indigenous populations [1–3, 9, 26], identified the needs of older Indigenous adults and their caregivers [8, 27, 28], and included the rates of unmet assistance needs among older Indigenous populations [6]. Data collection methods included one-on-one interviews [1–3, 6, 8, 9, 26, 28] and focus groups [8, 9, 26]. Eight of the studies included purposive sampling [1–3, 8, 9, 26–28] and one used random sampling [6]. Analyses methods included thematic [1–3, 8, 9, 26–28], content [2, 8, 9], descriptive [6, 8, 9], and grounded theory [28].

**Table 1:** Overview and description of included studies

First Author and Year Published	Continent	Inclusion Criteria	Study Design	Study Aim	Data Collection Methods	Sampling Strategy	Analysis Methods Used	Sample size (n) and mean age (age range)
Aspin et al. (2012) [2]	Australia	Participants who had a chronic illness AND/OR Care for a family member with chronic illness. Examine the experiences of people living with at least one of three index conditions (diabetes, chronic obstructive pulmonary disease, or chronic heart failure)	Qualitative	Focusing on Indigenous communities in Australia, this study reports on the perspectives of Aboriginal and Torres Strait Islander people affected by chronic illness and identifies approaches to combat the complexities associated with the increasing number of chronic illness.	One to one interviews	Purposive	Thematic Content	*n* = 19 34–70 years of age
Bell et al. (2015) [26]	Australia	It is evident that explicit criteria were not used to gather participants. However, if individuals expressed an interest in participating on the day of the focus group and completed the consent form then they could partake. Assessors included had a mostly Aboriginal client base and were stationed in remote communities or in Alice Springs	Qualitative	To examine the process of aged-care needs assessment for Aboriginal people in remote central Australia which then will be used to develop appropriate models of aged care.	One to one interviews Focus Groups	Purposive	Thematic	*n* = 11 aged-care assessors 7 community members Age not reported.
Browne et al. (2014) [27]	North America	For kūpuna groups: Native Hawaiian (self-identified), 60 years of age and older, cognitively alert, and willing/able to participate in a 1.5 h meeting. For ‘ohana caregiver groups: Native Hawaiian (self-identified) and providing unpaid care to an elder family member in the past 12 months	Qualitative	Examining the health needs and care preferences of community-dwelling nã-kūpuna (elders) and ‘ohana (family) caregivers in Hawai’i to make practice and policy recommendations	Focus groups	Purposive	Thematic	*n* = 24 kūpuna (elders) *n* = 17 o’hana caregivers Elders: 60–94 years of age (mean age 77) Caregivers: 38–77 (mean age 57)
Davis (2010) [3]	North America	Elders: self-identified as Native Hawaiian (full or part Hawaiian), aged > 60 years, who were currently living in Hawaii, noninstitutionalized, managing one or more chronic illness, and willing to participate in the study. Health care providers: self-identified as Native Hawaiian (full or part Hawaiian), were currently living in Hawaii, had experience in the health care field caring for the Native Hawaiian population, and were willing to share their ideas and perspectives.	Qualitative	This study aimed to explore the perspectives of Native Hawaiian kūpuna residing in Hawaii who live with chronic illness, in terms of gathering their perceptions, experiences and meanings of care. The goal of this study was to develop a deeper understanding of the care needs of Native Hawaiian kūpuna, and use that information to guide nurses in education (specifically, in providing culturally competent care).	One to one interviews (including photographs and field notes)	Purposive	Thematic	*n* = 15 kūpuna (elders) *n* = 5 health care providers Elders: 60 to 93 years of age (mean age 74.6) Health care providers: 40 to 60 years of age.
Habjan et al. (2012) [9]	North America	Individuals from each of the 13 First Nations communities were selected based on caregiving experience. Respondents represented perspectives of: (a) Chief and Council; (b) health care providers; (c) Elder; and (d) community members who were 18 years of age or older and had experience providing care to elderly individuals.	Qualitative	To explore the perspectives and experiences of elderly members of 13 rural and remote First Nations communities in northwestern, Ontario.	One to one interviews Focus groups	Purposive	Descriptive Thematic Content	*n* = 260 participants (216 completed surveys) 18 to 61 years of age and older.
Lowell et al. (2012) [28]	Australia	Must be either: Yolngu staff, community members (caregivers or people with chronic illness) or non-Yolngu staff involved with care for chronic conditions	Qualitative	The aim of this study was to work in collaboration with Aboriginal people to identity the limitations in current health care practice and to identify improvement strategies for means of communication pertinent to chronic illness.	One to one interviews (including videotapes of interactions between clients and health staff)	Purposive	Grounded Theory Thematic	*n* = 33 Yolngu health staff, clients, and other interested community members, and 8 health staff. 40 to 65 years of age.
Schure et al. (2015) [6]	North America	Participants were enrolled tribal members, who were over 55 years old, residing in the tribe’s service area, noninstitutionalized, and passed a cognitive screen.	Cross-sectional	The objective was to explore the prevalence and associated correlates of unmet assistance needs for aging American Indians.	One to one interviews	Random	Descriptive Statistical- demographics and characteristics only	*n* = 505 Age groups: 55 to 64, 65 to 74, and ≥ 75
Ward et al. (2011) [8]	Australia	Eligibility: individuals with one or more of the three conditions [type 2 diabetes (‘diabetes’), chronic obstructive pulmonary disease (COPD), and chronic heart failure (CHF)] aged between 30 and 85 years.	Qualitative	The aim was to engage individuals’ living with chronic illness to gather information and report on the way family members offer support, self-management behaviour and more broadly, coping with a chronic illness.	One to one interviews Focus groups	Purposive	Descriptive Thematic Content	*n* = 16 Indigenous and Torres Strait Islander Participants who had an illness, and 3 who cared for a family member with an illness 34 to 74 years of age.
Waugh et al. (2011) [1]	Australia	Participants were Indigenous Australians aged 45 years and above who were comfortable speaking in English.	Qualitative	The aim of this study was to investigate the views of older Indigenous peoples on what they believe contributes to their health and wellbeing as they age.	One to one interviews (through the use of storytelling)	Purposive	Thematic	*n* = 6 48 to 70 years of age (mean age of 59)

### Details on study participants

The age range of the participants was reported in eight studies, as presented in Table 1 [1–3, 6, 8, 9, 27, 28]. However, four studies did not differentiate between older adults, caregivers, and health care providers when reporting the age range [2, 8, 9, 28]. The mean age of older adults was reported in three studies [1, 3, 27].

Additional information on older adults as study participants is presented in Table 2. Of the nine articles included in the review, six reported the number of female participants (all of which had a female participation rate above 50%) [1, 3, 6, 9, 27, 28]. Two studies had a female participation rate above 75% [6, 28] and three studies did not report the number of females who participated [2, 8, 26]. Six of the nine studies explored MCC and included some exploration of ≥3 diseases [1–3, 6, 8, 28]. The most common chronic conditions reported were diabetes mellitus and cardiovascular disease [1–3, 6, 8, 28], cancer [1, 3, 6, 28], kidney disease [1, 6, 28], and hypertension [1, 3, 6]. Two studies set a requirement for participants that outlined number of chronic conditions needed, ranging from one or more [28] to a minimum of two [3]. It is to be noted that only one study reported ‘mental illness’ as a chronic condition [28]. One study did not explicitly state the ethnicity of their participants but did report that the research exclusively engaged 13 First Nations communities and was conducted in Treaty #3 Territory located in northwestern Ontario [9]. The remaining eight studies report ethnic identities of study participants as Aboriginal and Torres Strait Islander [2], Indigenous Australian [1], Aboriginal [26], Native Hawai’ian [3, 27], Yolngu (Aboriginal people in northern territory of Australia) [28], American Indians [6] and Indigenous [8]. Two studies reported the living situation or marital status of participants and found that between 20.5 to 70% were married [3, 6]; 80% widowed [3]; and 38% single [6]. Education was reported by three studies where between 5 and 28% of participants had either no formal education or less than grade 12, 25 to 80% had completed high school or General Education Development (GED), and 10 to 37% had either a college- or graduate-level education [3, 6, 27]. Education was not reported in six of the studies. One noted that many Yolngu are fluent in multiple Aboriginal languages, despite their competency in English ranging from minimal-to-high [28].

**Table 2:** Details on older adults study participants

First Author and Year Published	Percentage of Female	Diseases	Number of Diseases	Ethnicity	Socio-economic Status (SES)	Living Situation/ Marital Status	Education	Other
Aspin et al. (2012) [2]	NR	Diabetes mellitus Chronic Obstructive Pulmonary Disease (COPD) Chronic Heart Failure	NR	Aboriginal (Australia) and Torres Strait Islander	NR	NR	NR	–
Bell et al. (2015) [26]	NR	NR	NR	Aboriginal	NR	NR	NR	–
Browne et al. (2014) [27]	65%	NR	NR	Native Hawai’ian	NR	NR	15 (63%) graduated from high school or earned a GED, and eight (37%) had some college	Elders voiced an appreciation for receiving help with household chores, shopping and appointments/visits to doctor. Few are reported as needing help bathing, dressing, eating, or getting in and out of bed.
Davis (2010) [3]	53%	Diabetes mellitus Rheumatic arthritis Cancer Heart disease [hypertension, heart attack and/or stroke] Asthma Osteoporosis	minimum: two chronic conditions with varying acuity levels	Native Hawai’ian	NR	widowed: 7 married: 8	1 no formal education, 80% high school diploma, 2 with some graduate	–
Habjan et al. (2012) [9]	54%	NR	NR	Not explicitly reported, but research was conducted in First Nations area (Treaty #3)	NR	NR	NR	–
Lowell et al. (2012) [28]	76%	Diabetes mellitus Cancer Chronic kidney disease Cardiovascular disease Chronic airways disease Mental illness	One or more	Yolngu- Aboriginal people of Australia	NR	NR	Not reported. It is noted that while Yolngu are fluent in, what is often, a range of Aboriginal languages. Competency in oral English has been reported to range from high-to-minimal.	It is common for Yolngu not to speak English as their first language
Schure et al. (2015) [6]	77.9%	Diabetes mellitus Cancer Heart disease stroke, Angina Congestive heart failure Heart attack Lung disease Parkinson’s disease High blood pressure Kidney disease Liver disease	NR	American Indians	NR	single: 192 married: 104	≥ Grade 12: 141; and < Grade 12: 144	NR
Ward et al. (2011) [8]	NR	Diabetes Mellitus Chronic Obstructive Pulmonary Disease Chronic heart failure	NR	Indigenous	NR	NR	NR	NR
Waugh et al. (2011) [1]	66%	Diabetes mellitus Rheumatic arthritis Cancer Cardiovascular disease Emphysema Asthma Obesity Kidney disease High blood pressure High cholesterol	NR	Indigenous Australians	NR	NR	NR	NR

*N.R*. Not reported

#### Health professional characteristics

Table 3 outlines the characteristics of the health professionals and caregivers that participated in the studies. Five of the articles did not include health professionals within their studies [1, 2, 6, 8, 27], while the other studies highlighted a range of providers including aged-care specialists [26], nurses [3, 9], community health representatives [9, 28], community wellness workers [9] and healers [3], among others. Only one study reported the years of experience of the health professionals who participated, and they ranged from 6 months to 20 years [26]. Additionally, in one study education level of the health professionals was reported as varied, with one having a high school diploma, one with an associate degree, and three having graduate degrees [3].

**Table 3:** Details on caregiver and health care provider study participants

Health care provider (hcp) characteristics						
First Author and Year Published	Type of provider		Years of Experience		Other	
Aspin et al. (2012) [2]	HCP not included/not applicable		–		–	
Bell et al. (2015) [26]	Specialty: Aged Care		6 months to 20 years			
Browne et al. (2014) [27]	HCP not included/not applicable		–		–	
Davis (2010) [3]	Two nursing faculty, a health care manager, a clinic outreach worker, and a Native Hawaiian healer.		NR		40 to 60 years of age. All Native Hawai’ian and living in Hawai’i. One had a high school diploma, one an associate’s degree, and three had graduate degrees.	
Habjan et al. (2012) [9]	Community health representatives, home and community care coordinators, long-term care coordinators, home support workers, community wellness workers, nurses, homemakers, health directors, and diabetes educators.		NR			
Lowell et al. (2012) [28]	Community Health staff		NR		There were 8 non-Yolngu health staff interviewed. None of the nursing, medical, or allied health staff share a common cultural understanding with their Yolngu clients. In addition to the language barriers between health staff and Yolngu clients, there is also a high turnover rate of Balanda health staff.	
Schure et al. (2015) [6]	HCP not included/not applicable		–		–	
Ward et al. (2011) [8]	HCP not included/not applicable		–		–	
Waugh et al. (2011)	HCP not included/not applicable		–		–	
CAREGIVER (CG) CHARACTERISTICS						
First Author and Year Published	Age	% Female	Relationship to Patient	Education	Health	Other
Aspin et al. (2012) [2]	CG not included/not applicable	–	–	–	–	–
Bell et al. (2015) [26]	CG not included/not applicable	–	–	–	–	–
Browne et al. (2014) [27]	38–77 years, mean 57	65%	The study reports the incidence of ‘ohana members caring for parents (8), a spouse (7), sibling (2), uncle (1), grandparent (1) or cousin (1).	11 had completed high school, and 6 had some college.	Not reported	Caregivers report that they provide care anywhere between 2 to 24 h a day. Most often, they are assisting elders with shopping, visiting the doctor’s office, household chores (cleaning, cooking) and other personal tasks (bathing, medications and paying bills). Providing care for an average of 7 years (ranging from 3 to 10 years), almost half of the ‘ohana caregivers (7/16) were caring for multiple elders at the same time.
Davis (2010) [3]	CG not included/not applicable	–	–	–	–	–
Habjan et al. (2012) [9]	CG were included in the study but it was not clarified.	NR	NR	NR	NR	NR
Lowell et al. (2012) [28]	NR	NR	NR	Oral English competence ranges from high-to-minimal, and literacy in any language is often limited in this predominantly oral culture	NR	NR
Schure et al. (2015) [6]	CG not included/not applicable	–	–	–	–	–
Ward et al. (2011) [8]	CG were included in the study but it was not clarified.	NR	Family Member	NR	NR	NR
Waugh et al. (2011) [1]	CG not included/not applicable	–	–	–	–	–

*NR* Not reported, − = not applicable

#### Family/friend caregiver characteristics

Caregivers were not included in five of the studies [1–3, 6, 26], with two of the studies including caregivers where their details such as age, gender, relationship to patient, and education were unclear [8, 9]. One study reported the age of the caregivers (38–77 years, m = 57) and the percent of female caregivers (65%) [27]. Two studies reported the relationship between the caregiver and the patient, while the specificities in labels such as caring for a ‘family member,’ ‘parents,’ ‘spouse,’ or ‘sibling,’ varied [8, 27].

### Quality assessment

All of the papers were assessed based on the Mixed Methods Appraisal Tool (see Table S2) [25]. Of the eight qualitative studies, two did not have a clear process outlined for analyzing qualitative data that were relevant to answering the research questions [3, 26]. Additionally, of the qualitative studies, little consideration was given to how the findings related to the researchers’ influence or the interaction with participants [2, 3, 8, 9, 26, 27]. All studies had a clear research question or objective and collected data that was relevant to address those inquiries.

The cross-sectional study [6] was appraised based on the MMAT guidelines for quantitative non-randomized studies [25]. Participant recruitment was done by random sampling to minimize selection bias. It is evident that the research team used the appropriate measurements to assess the relationship between the exposure (residential location) and outcomes (unmet assistance need with respect to activities of daily living and instrumental activities of daily living). Additionally, the researchers accounted for differences between groups and participants. However, it remains unclear whether the outcome data was complete or if there was an acceptable response rate.

### Research approaches used in studies

Data collection methods used in the studies primarily includes one-on-one interviews [1–3, 6, 8, 9, 26, 28] and focus groups [8, 9, 26]. Culturally informed methods of data collection were reflected in three studies [1, 26, 27]. Storytelling, an ethically appropriate and culturally sensitive data collection method, was observed as a semi-structure interview that allowed participants to expand on key points and encouraged dialogue with the interviewer [1]. A separate culturally informed method, a listening study format, encouraged elders to navigate their own inquiry by being allowed more time to answer a series of questions [26]. Using semi-structured interviews, all participants of one study were given the opportunity to engage with Yolngu researchers and interpreters to use their preferred language [27], a culturally appropriate method of data collection.

### Overview of proximal, intermediate, and distal determinants of health

Primarily, we applied the IDH framework to examine the needs of older adults and caregivers and this was supplemented by an understanding of the ICF framework. Proximal determinants of health were addressed within all the studies (see Table 4). Most notable of the proximal determinants addressed were education and literacy [1–3, 6, 8, 9, 27, 28], ethnicity [1–3, 6, 8, 26–28], social support networks [1–3, 6, 8, 9, 26, 27], and access issues [1–3, 9, 26–28]. A small number of studies addressed socioeconomic status [1, 3, 9, 27], living circumstances [3, 9, 26, 27], and food security [1–3, 27]. A smaller number of studies addressed language [9, 26, 28], living situation [6, 9], and early childhood development [1, 2]. In contrast, five proximal determinants were not addressed in relation to the health and social care needs of older Indigenous adults with MCC: gender, social capital, social isolation, marital status, and relationship to patient.

**Table 4:** Overview of proximal determinants that were identified in the discussion of health and social care needs

First Author and Year Published	SES	Gender	Language	Education and Literacy	Ethnicity	Living Circumstances (urban/rural)	Living Situation (alone or not)	Social Capital and Social Isolation	Social Support/ Network(s)	Marital Status	Relationship to Patient	Access Issues	Food Security	Early Childhood Develop.
Aspin et al. (2012) [2]				X	X				X			X	X	X
Bell et al. (2015) [26]			X		X	X			X			X		
Browne et al. (2014) [27]	X			X	X	X			X			X	X	
Davis (2010) [3]	X			X	X	X			X			X	X	
Habjan et al. (2012) [9]	X		X	X		X	X		X			X		
Lowell et al. (2012) [28]			X	X	X							X		
Schure et al. (2015) [6]				X	X		X		X					
Ward et al. (2011) [8]				X	X				X					
Waugh et al. (2011) [1]	X			X	X				X			X	X	X

Intermediate determinants of health were addressed within all studies but one [See Table 5]. Six studies addressed health promotion and health care [2, 8, 9, 26–28]. A small number of studies addressed ways of knowing [1, 3, 28], kinship networks [2, 9], relationship to land [26] and government and private enterprises [8].

**Table 5:** Overview of intermediate determinants that were identified in the discussion of health and social care needs

First Author and Year Published	Health Promotion and Health Care	Government and Private Enterprise	Kinship Networks	Relationship to Land	Ways of Knowing
Aspin et al. (2012) [2]	X		X		
Bell et al. (2015) [26]	X			X	
Browne et al. (2014) [27]	X				
Davis (2010) [3]					X
Habjan et al. (2012) [9]	X		X		
Lowell et al. (2012) [28]	X				X
Schure et al. (2015) [6]					
Ward et al. (2011) [8]	X	X			
Waugh et al. (2011) [1]					X

Six of the nine articles discussed distal determinants of health as affecting health and social care needs of older Indigenous adults with MCC (See Table 6) which included historical (i.e. colonization, oppression, assimilation, interrupted access to land) [1–3, 26, 27] and contemporary (i.e. social, political, economic) [1–3, 9, 27, 28]. The spiritual dimension of distal determinants are intimately connected to the proximal (i.e. culture) and intermediate (i.e. Indigenous worldviews, self-determination) determinants.

**Table 6:** Overview of distal determinants that were identified in the discussion of health and social care needs

First Author and Year Published	Historical (i.e. colonization, oppression, assimilation, interrupted access to land)	Contemporary Structures (i.e. social, political, economic)
Aspin et al. (2012) [2]	X	X
Bell et al. (2015) [26]		
Browne et al. (2014) [27]	X	X
Davis (2010) [3]	X	X
Habjan et al. (2012) [9]		X
Lowell et al. (2012) [28]	X	X
Schure et al. (2015) [6]		
Ward et al. (2011) [8]		
Waugh et al. (2011) [1]	X	

### Summary of needs identified within the studies

Five themes emerged from the data (see Table 7) and were consistent between older adults, caregiver and health care provider perspectives. The resulting themes outline the need for: 1) The need for accessible health services; 2) The need for building community capacity; 3) The need for improved social support networks; 4) The need for preservation of cultural values in health care; and 5) The need for wellness-based approaches.

**Table 7:** Overview of needs identified in the studies

First Author and Year Published	Needs identified by Older adults/Caregivers/Health Care Providers/Other
Aspin et al. (2012) [2]	An established and visible Aboriginal and Torres Strait Islander healthcare professional workforce. Acknowledgment of the important roles that family members and peers have in assisting the management of chronic illness. Patients also want to be actively involved in the problem-solving processes when it comes to their chronic illness. Long term relationships with care providers.
Bell et al. (2015) [26]	1. Cultural perspectives on aged care: relationships, family participation, aging at home, staying on country, wellness/cultural identity. 2. Context of Service Delivery: staffing housing, living conditions and hardship and carer burden. 3. Equity and access to services: barriers to service entry, poor communication and accommodation of cultural expectations. 4. Program (mis)alignments. Assessment at high level of care needs often doesn’t match the basic service provided. The unpaid care-work associated with caring for a family member often occurs outside of work hours fusing together work roles and family responsibilities. Building capacity in communities is needed to provide sustainable levels of care and service delivery that will adequately address the needs of older Aboriginal people. There is also a need to restrict and reduce the amount of hardships experienced be carers and older people that would allow them to continue living ‘on country’.
Browne et al. (2014) [27]	Importance of cultural values in service design and delivery (diet, prayer and spiritualty, staff trained in cultural competence), caregiving as cultural preservation for family and community, specific health worries, increased frailty). Importance of cultural values in service design and delivery (diet, prayer and spiritualty, staff trained in cultural competence), caregiving as cultural preservation for family and community, family support programs (caregiver education, respite services, transportation). Caregiving is viewed as both a shared responsibility of the family unit and government system.
Davis (2010) [3]	Kupuna want nurses to respect their cultural heritage, honour their past and understand their worldview and values. It is also reported that Kupuna want nurses to be interested in their family, include Hawaiian foods and healing practices as a part of their care-plan, allow for their voices to be heard (listening and asking questions) and provide care that is friendly and personal (not overtly direct). They want information that is straightforward and given with patience. They also want care to be provided in environments that feel comfortable and safe. Overall, Kupuna want a health care system that not only reflects their culture and values but offers programs and services that are specific to the needs of the ‘ohana
Habjan et al. (2012) [9]	With the family members being unavailable to provide care, there is an increasing dependence on paid community caregivers. However, limited health human resources create a heavy reliance on a very few people. Contrast to traditional Elder-youth relationships, Elders now feel that young people do not offer the help needed or respect. Elders also need greater access to health services within community. It is also reported that cultural sensitivity training is needed for health care professionals and support personnel. Specifically, improvement in cultural and spiritual awareness, understanding and respect that align with First Nations traditional teachings and language. There is a need for psychological support training in the areas of grief support, counselling and crisis management. In addition, caregivers also want to learn how to identify depression symptoms and implement proper self-care (relaxation therapy and stress management). Participants outlined that home support training is needed for both health care providers and family, including personal support workers and homemaking training. Participants also identified that they want to stay in community to access health care, therefore enhanced training is required to meet this demand. Due to the lack of employment opportunities and the associated economic constraints, many young people look for work outside the community. This path takes family members away from the elders that need them to provide care. There is an immense need for a return to the traditional ways of intergenerational family caregiving. It was also reported that personal support workers are needed as well as, increased opportunities to receive financial aid for travel, food and medical expenses.
Lowell et al. (2012) [28]	Yolngu require access to meaningful information that would facilitate informed decision making related to the prevention and management of chronic disease. There is also a need for medical and physiological terminology to not be assumed as common knowledge by HPs when explaining to clients about their chronic diseases, their causes, consequences and management. Some Yolngu also voiced the concern that it appears that important information about their condition was being withheld from them and their families.
Schure et al. (2015) [6]	There is the potential for the results to exhibit bias due to American Indians being more reluctant to report needing assistance than other racialized groups.
Ward et al. (2011) [8]	The importance of community, family and ‘yarning’ is expressed as a significant source of support and knowledge. Exchanging stories about the realities of living with a chronic illness is described as having a ‘yarn’ and assists in relaxing persons living with chronic illness. It is also outlined that family should be cautious of providing unsolicited practical support. The priority being to ensure that those managing a chronic illness maintain independence, while simultaneously normalising support through reciprocal care spanning the life-cycle. In hopes of increasing good health outcomes, informal carers need support that would offload the exhausting and demanding nature of caring for family and community members.
Waugh et al. (2011) [1]	There is an expression of anger and frustration by participants because of the contemporary attitudes (uncaring) toward older people. Aboriginal Medical Services (AMS) are important because they make health care accessible and provide opportunities for Aboriginal people to socialize and connect to both each other and their culture. Groups are also identified as essential for developing friendships, making space for participants to share about their struggles and reduce social isolation. It is also identified that asking for help needs to be an empowering experience rather than something that leads to the development of a negative self-identity.

#### The need for accessible health services

Many of the participants in the studies spoke of the lack of access to services in their communities [1, 9, 27]. Accessible health services require that services are reasonably and conveniently available but also that they are culturally relevant to the community they serve, as discussed further in theme 4 below. Several older adults commented on how the availability of services varied in each community [9] and expressed the need for more services that would allow them to remain in their homes [27]. In addition, the health centres that were available provided services on average from 1 day a month to several days a week with no services after business hours or on weekends [9], which was less than optimal. All these circumstances have led to unmet needs.

When services were readily available, access to the information needed about chronic conditions was perceived as a barrier. Some older adults felt that important information was being withheld from them [28]. Older adults and their caregivers spoke about a history of negative prejudicial treatment, such as patronizing language and discrimination, that led to poor communication and uptake of recommendations from health professionals [2, 27]. Access to meaningful information [28] that is straightforward and given with patience and care [3] is what some participants required as well as learning how to survive within a dominant Western culture [1, 3].

Participants also expressed that access to services was highly dependent on geographical, infrastructural, and financial support available to the communities [2, 9, 26, 27]. Transportation was available in some communities; however, access to services was limited due to geographical isolation, financial constraint, inadequate vehicles and the lack of drivers [9]. Additional financial resources were required for participants to afford the food recommendations, medical expenses to manage their conditions [9], and to help offset the reliance on family carers [8]. This cost however was sometimes picked up by other local community funding, which unfairly burdened the community’s financial status [9].

#### The need for building community capacity

A clear overall need to build community capacity was derived from sub-themes of education, training and knowledge. The literature suggested that community members want to stay in their local community when accessing services. To be able to meet that preference, there is a need for education, support, and training for both health care providers and informal family carers [9, 26]. The lack of training and support has resulted in community members and care staff being overburdened and required to take on roles for which they are not trained [8, 9]. Indigenous community members value and rely on family and local community support. Unpaid caregiving often occurs outside of work hours and led to hardship for some including, caregiver burden, stress [26], and financial strain [27]. In addition, accepting help was sometimes culturally difficult for older adults [27].

Lack of services and resources in communities often require people to relocate and there is a concern that these community networks will be lost [8]. Overall, community members feel there is a lack of capacity within communities to effectively support the health care needs of Indigenous community members.

#### The need for improved social support networks

The need for improved social support was highlighted in many of the articles [1–3, 8, 9, 26, 27]. Many of the older adults outlined the importance of being care for at home by their family members and friends [3]. Remaining on their homelands was also of significant importance to older Indigenous peoples [26].

Studies reported the need for a return to the traditional ways of intergenerational caregiving [9] because the responsibility of caregiving in families was also seen as a way to preserve culture [27]. Children were seen as playing an important role as advocates to support elders with chronic illness [2] yet sometimes elders were reluctant to burden their children [27]. Grandchildren were viewed as a motivating factor to remaining in good health in order to fulfill the grandparenting role which was a great source of happiness and satisfaction [8]. One study reported that informal paid caregivers are required to off-load the demanding nature of care being provided by family and community members [8]. Another noted that the traditional extended family system was changing [9], such that family and community networks were breaking down [9]. There was an obvious need for shared responsibility of social support (caregiving) by the family unit and government system [27]. The critical role that family members, peers and social networks have in assisting with the management of the elders’ chronic condition was also highlighted [2].

It was suggested that the opportunity for educational interventions be provided to the caregivers to assist with mitigating anxiety and cultivating supportive practices [8]. In addition, training for staff providing home support was recommended [9]. Finally, creating social avenues to enable older adults to connect with both each other, and their culture, through health promoting activities was seen as important [1].

#### The need for preservation of cultural values in health care

To fulfill their cultural roles, elders require support. They need support to advocate for the younger generations [1]; educate others about health and well-being, including how to manage chronic illnesses [2]; and interact with family, especially children [2] to motivate themselves to remain healthy, happy, and satisfied [1]. There was an emphasis on Indigenous values of health. Hawaiian informants value “pono (harmony, doing right), mana (strength, energy, spirituality), ‘ohana (extended family)”, and ha’aha’a (humility and respect)” [3]; and equate eating healthy with culture and diet [3, 27]. For example, there is an importance of “Taro and Poi for physical, emotional, and spiritual health” [27]. Health is viewed as holistic (physical, social, mental, spiritual [1]); circular, and is interconnected with a spiritual unity between the “land, our ancestors, family, and a higher power” [3]. Staying ‘on country’ to age-at-home or to die on one’s homeland was highlighted as a cultural requirement for Australian Aboriginals [26]. Ultimately, intergenerational family caregiving is described as a means of cultural preservation [9, 27].

Community members discussed the need for health care services to be culturally responsive and incorporate Indigenous ways of knowing and traditional approaches to health and wellness [9], reflecting cultural competency. Older persons also spoke about the need for staff to have culturally competent training in order to provide the care they needed [9, 26, 28]. Providing trained interpreters in situations where consent was required (like surgery) was also difficult to find [28] as there was a lack of availability of staff who speak the native languages of the First Nations communities [9]. Health care professionals can respect cultural heritage and identity by honouring and understanding the differing worldviews and values of varying Indigenous groups [3] through service design and delivery [27], and by providing social avenues to connect individuals with their culture through health promotion activities [1]. Respect can also be expressed by health care providers and support personnel through improvements in cultural and spiritual awareness and understanding by taking cultural sensitivity training [9]; and by having humble, caring approaches through establishing a personal connection, being friendly and personable, and having a sincere concern for the “Kupana and his/her ‘ohana” [3].

#### The need for wellness-based approaches

Wellness emerged through two sub-themes: self-care and wellness-driven activities. There is a need for older adults and caregivers to be actively involved in self-care by: acquiring knowledge and skills associated with implementing self-care approaches such as relaxation and stress management [9]; accessing grief support and resources to reduce emotional and financial burden [27]; and accessing social and group support networks to gather and share experiences, connecting with each other and their culture [1]. Furthermore, caregivers need to receive psychological training in the area of grief support, counselling, and crisis management [9]. In addition, there is a need for education to: increase both Western and Traditional knowledges, awareness, and understandings about the impact of behavioural related factors (e.g. smoking, diet, physical inactivity) on the development of chronic conditions such as depression [9, 28]; to facilitate informed decision making related to the prevention, treatment and management of chronic diseases [2, 28]; and to understand the negative effects of chronic health conditions on overall health outcomes [28]. These activities promote self-care of both the caregivers and older adults.

### Health stakeholder discussion

The workshop participants reviewed the methods for the scoping review and the themes that emerged. Overall, they confirmed that the themes captured the health and social care needs of Indigenous older adults with multiple chronic conditions and their caregivers. They encouraged the review team to move beyond description and work toward local application of international review findings. The participants discussed the term “Indigenous” and highlighted that the international nature of the scoping review may have diluted the importance of place and nationhood. They emphasized the importance of retaining the integrity and names of individual nations and tribes. A great example of this is how researchers clearly outline First Nations communities in northwestern Ontario [8] – where the specific group of Indigenous people (First Nation) and location is identified.

Participants provided an in-depth interpretation of what “accessible health services” means. In particular, the group had an in-depth discussion about the underlying thread of cultural safety and trauma-informed care. Cultural safety, a concept originated by Dr. Irihapeti Ramsden in Aotearoa/New Zealand, seeks to address structural inequalities in health care delivery experienced by Indigenous peoples [29]. The group felt that cultural safety was an important concept but that, in the local context of First Nations health care experiences in Ontario, it is widely misunderstood and used as a “buzzword” without meaning. The group emphasized that the important part of cultural safety is to create an inclusive, neutral space where human-to-human interaction is comfortable and safe. They also highlighted that a mis-application of the concept could result in people feeling more disconnected from their care if they do not wish to receive culturally-specific care. This reflects the perspectives of a group of First Nations people with strong connection to First Nations health care policy, research and services. We do not know whether this perspective would have been different among a different group of First Nations people living with multiple chronic conditions.

A second area of focus was the importance of integration, coordination, and navigation within healthcare, particularly related to specialist services, and between healthcare and other sectors. This is particularly important in the context of First Nations-based health and social services because there is a mix of federal, provincial and First Nations funding, accountability, and jurisdiction in the services provided. The group stressed the importance of planning regionally to develop strong case management practices to support older people and their caregivers. They suggested that standardized procedures and communication could be developed, implemented and evaluated to improve the integration, and ultimately the accessibility, of health services for First Nations people in Ontario.

The group emphasized that the key impact of the scoping review is that it provides a foundation for determining how the themes relate to local priorities and recommendations. Those gathered at the workshop were looking for actionable and applied recommendations to come out of the work that could be applied at a local level.

## Discussion

This scoping review provides the first informative overview of health and social support needs, priorities, and preferences for a globally aging Indigenous population who are living outside of long-term care settings with multiple chronic conditions and their caregivers.. Our study highlighted the need for access to services and information, building community capacity, social support, preservation of cultural values in health care, and wellness approaches. Similar themes were found in the broader review of the literature that focused on non-Indigenous older adults such as access to information and a focus for prevention strategies (i.e. wellness approaches) to better assist older adults to manage their conditions [16].

Indigenous populations have a strong focus on social support which mostly includes family involvement and the need to return to the traditional ways of intergenerational caregiving [1, 9, 27]. Additionally, staff caring for older adults were called on to be trained in cultural and spiritual awareness, and to develop an understanding that aligns with Indigenous traditional teachings and language [9]. While non-Indigenous older adults were concerned with coordination of services and supports [16], Indigenous populations were heavily concerned with health and social support access issues [1–3, 9, 26–28], due to the remote locations to which many of them reside and the limited availability of clinics open to meet their needs [9]. Finally, unique to research conducted with Indigenous older adults with MCC was a focus on building community capacity [8, 9, 26] and the need for preservation of cultural values in health care [1–3, 26, 27].

Wellness-driven activities include an incorporation of cultural perspectives about relationships, family participation, aging at home, staying on land, wellness/cultural identity on aged care [26]; connecting to culture, spirituality, and balance [3, 26]; and engaging with elders to disseminate knowledge and information [2]. There is a need to incorporate Indigenous concepts of health (including those represented within the Medicine Wheel) into health care service delivery [9]. For example, in Canada, Indigenous health is perceived holistically and includes physical, mental, emotional, spiritual, and cultural aspects of life [9]. There is a need to apply a systems perspective as a useful approach to understand the health and well-being of the Indigenous older population [9].

The use of the Indigenous Determinants of Health (IDH) theoretical framework [10, 11], and the International Classification of Functioning, Disability and Health (ICF) framework provided a unique perspective to understanding the health and social care needs of older Indigenous adults. Combining the frameworks and prioritizing the IDH in the presentation of the results offers a holistic understanding of the preferences of Indigenous older adults as related to wellbeing and quality of life on overall functioning. Although the ICF framework provided us with an understanding that individual, environmental, and personal factors are interrelated, future studies on Indigenous health and social care needs may wish to focus on the IDH theoretical framework only. The IDH when used as a theoretical framework strengthened this study by helping us recognize the importance of Indigenous values such as relationships, kinship, language, and cultural traditions and how they impact individual health. The key IDH that influenced older adults’ needs were education and literacy, ethnicity, and social support/network (proximal); health promotion and health care (intermediate); and a combination of historical and contemporary structures (distal).

The strengths of this review included a comprehensive search of electronic data bases carried out by expert health sciences librarians, which involved some exploration on how to determine search terms for “Indigenous populations,” and two Indigenous reviewers simultaneously abstracting the data and completing multiple checks of the articles in this review. However, a limitation to the search strategy exists in that the database search did not include social programs for Indigenous people that would not be in the health literature. Extending the search to include this will further provide confidence in the representation of the findings related to older Indigenous adults living with MCC.

The IDH and ICF frameworks served as a guide to present our findings and the results resonated with our stakeholder group, which helped to validate the results. Although we incorporate spirituality in the theme “the need for cultural preservation in health care, it must be analyzed more clearly and with more vigor than this analysis has attempted. Further studies that incorporate the IDH model as a theoretical framework must pay closer attention to the importance of spirituality in the analysis. The Indigenous spirituality is a core value and central to balancing holistic health; and is considered as equal as physical, mental and emotional health [9, 27]. Although we did not explicitly examine spirituality as a separate determinant of health, it is reflected in the analysis of the distal determinants of health, specifically the historical and contemporary structures, including Indigenous worldviews.

## Conclusion

This scoping review was developed, overseen, and carried out in a collaborative partnership with a research team consisting of knowledge users, health stakeholders and Indigenous scholars. This research is important for health care professionals and leadership working with Indigenous older adults who seek to develop interventions that will meet these needs and assist with improving quality of life. The development of knowledge translation activities needs to reflect an all-inclusive approach between First Nations people and the health sector. Combined with key recommendations from health stakeholders, further thematic analysis of the articles using an approach that reflects local Indigenous knowledge and concepts such as accessible health services and cultural safety will improve findings. The First Nations-specific group will seek further opportunities to expand on the recommendations from this scoping review outside of publishing a manuscript.

## Supplementary information

**Additional file 1: Table S1.** Medline Search. Table of Medline Search

**Additional file 2: Table S2.** Quality Assessment Using the Mixed Methods Appraisal Tool. Table of nine studies assessed using the MMAT

## Data Availability

The datasets used and/or analyzed during the current study are available from the corresponding author upon request. All data and material will be made available.

## Abbreviations

AMS: Aboriginal Medical Services
CG: Caregiver
CHF: Congestive heart failure
COPD: Chronic obstructive pulmonary disease
GED: General Education Development
HCP: Health care provider
ICF: International Classification of Functioning, Disability and Health
IDH: Indigenous Determinants of Health
MMAT: Mixed Methods Appraisal Tool
MCC: Multiple chronic condition
NR: Not reported
PRISMA-P: Preferred Reporting Items for Systematic Reviews and Meta-Analyses Protocol
PRISMA: Preferred Reporting Items for Systematic Reviews and Meta-Analyses
PI: Principal Investigator
WHO: World Health Organization
