# Supplementary material for: Identifying and understanding the health and social care needs of Indigenous older adults with multiple chronic conditions and their caregivers: a scoping review
Source: BMC Geriatr. 2020 Apr 19;20:145. doi: 10.1186/s12877-020-01552-5 (PMC7168986; doi:10.1186/s12877-020-01552-5)
Supplement: Supplementary file 2 — Additional file 2: Table S2. Quality Assessment Using the Mixed Methods Appraisal Tool. Table of nine studies assessed using the MMAT [file 12877_2020_1552_MOESM2_ESM.docx]

**Table S2 Quality Assessment using the Mixed Methods Appraisal Tool (MMAT) ***

| **First Author and Year Published** | **Screening questions** | | **Qualitative** | | | | **Quantitative non-randomized** | | | |
| --- | --- | --- | --- | --- | --- | --- | --- | --- | --- | --- |
|  | Are there clear qualitative and quantitative research questions or a clear mixed-methods research question (objectives)? | Do the collected data address the research question (objective)? | 1.1 Are the sources of qualitative data relevant to address the research question (objective)? | 1.2 Is the process for analyzing qualitative data relevant to address the research question (objective)? | 1.3 Is appropriate consideration given to how findings relate to the context in which the data were collected? | 1.4 Is appropriate consideration given to how findings relate to researchers’ influence, e.g. through their interactions with participants? | 3.1 Are participants recruited in a way that minimizes selection bias? | 3.2 Are measurements appropriate regarding the exposure/intervention and outcomes? | 3.3 In the groups being compared, are the participants comparable, or do researchers take into account the difference between these groups? | 3. 4 Are there complete outcome data (80%) or above), and, when applicable, an acceptable response rates (60% or above), or an acceptable follow-up rate for cohort studies? |
| Aspin et al. (2012)^(6)^ | Yes | Yes | Yes | Yes | Yes | Can’t tell | - | - | - | - |
| Bell et al. (2015)^(22)^ | Yes | Yes | Yes | Can’t tell | Yes | Can’t tell | - | - | - | - |
| Browne et al. (2014)^(23)^ | Yes | Yes | Yes | Yes | Yes | Can’t tell | - | - | - | - |
| Davis (2010)^(2)^ | Yes | Yes | Yes | Can’t tell | Yes | Can’t tell | - | - | - | - |
| Habjan et al (2012)^(4)^ | Yes | Yes | Yes | Yes | Yes | Can’t tell | - | - | - | - |
| Lowell et al. (2012)^(24)^ | Yes | Yes | Yes | Yes | Yes | Yes | - | - | - | - |
| Schure et al. (2015) ^(8)^ | Yes | Yes | - | - | - | - | Yes | Yes | Yes | Can’t tell |
| Ward et al. (2011) ^(3)^ | Yes | Yes | Yes | Yes | Yes | No | - | - | - | - |
| Waugh et al. (2011)^(5)^ | Yes | Yes | Yes | Yes | Yes | Yes | - | - | - | - |

*As there was no study that had a quantitative randomized controlled trial, quantitative descriptive design, or mixed methods, the quality criteria of the MMAT for those 3 study designs were omitted from the table
